# Myocardial MiR-30 downregulation triggered by doxorubicin drives alterations in *β*-adrenergic signaling and enhances apoptosis

**DOI:** 10.1038/cddis.2015.89

**Published:** 2015-05-07

**Authors:** L Roca-Alonso, L Castellano, A Mills, A F Dabrowska, M B Sikkel, L Pellegrino, J Jacob, A E Frampton, J Krell, R C Coombes, S E Harding, A R Lyon, J Stebbing

**Affiliations:** 1 Division of Oncology, Department of Surgery and Cancer, 1st Floor, Imperial Centre for Translational and Experimental Medicine (ICTEM), Imperial College, Hammersmith Hospital, Du Cane Road, London W12 0NN, UK; 2 National Heart and Lung Institute, Imperial College, 4th Floor, ICTEM, Hammersmith Hospital, Du Cane Road, London W12 0NN, UK; 3 Hepato-Pancreato-Biliary Surgical Unit, Department of Surgery and Cancer, Imperial College, Hammersmith Hospital, Du Cane Road, London W12 0NN, UK; 4 NIHR Cardiovascular Biomedical Research Unit, Royal Brompton Hospital, Imperial college, London SW3 6NP, UK

## Abstract

The use of anthracyclines such as doxorubicin (DOX) has improved outcome in cancer patients, yet associated risks of cardiomyopathy have limited their clinical application. DOX-associated cardiotoxicity is frequently irreversible and typically progresses to heart failure (HF) but our understanding of molecular mechanisms underlying this and essential for development of cardioprotective strategies remains largely obscure. As microRNAs (miRNAs) have been shown to play potent regulatory roles in both cardiovascular disease and cancer, we investigated miRNA changes in DOX-induced HF and the alteration of cellular processes downstream. Myocardial miRNA profiling was performed after DOX-induced injury, either via acute application to isolated cardiomyocytes or via chronic exposure *in vivo*, and compared with miRNA profiles from remodeled hearts following myocardial infarction. The miR-30 family was downregulated in all three models. We describe here that miR-30 act regulating the *β*-adrenergic pathway, where preferential *β*1- and *β*2-adrenoceptor (*β*1AR and *β*2AR) direct inhibition is combined with Gi*α*-2 targeting for fine-tuning. Importantly, we show that miR-30 also target the pro-apoptotic gene *BNIP3L/NIX*. In aggregate, we demonstrate that high miR-30 levels are protective against DOX toxicity and correlate this in turn with lower reactive oxygen species generation. In addition, we identify GATA-6 as a mediator of DOX-associated reductions in miR-30 expression. In conclusion, we describe that DOX causes acute and sustained miR-30 downregulation in cardiomyocytes via GATA-6. miR-30 overexpression protects cardiac cells from DOX-induced apoptosis, and its maintenance represents a potential cardioprotective and anti-tumorigenic strategy for anthracyclines.

Doxorubicin (DOX) is the most used anthracycline in the oncology clinic, delivering anti-tumor activity against numerous types of malignancy.^1, 2^ The exact mechanisms accounting for the anti-cancer action(s) of anthracyclines remain poorly understood, although a variety of effects have been proposed, most notably inhibition of topoisomerase II (Top2).^2^ Patients receiving DOX treatment often experience adverse effects, the most serious being dose-dependent cardiotoxicity that can lead to heart failure (HF). Efficient prevention and early diagnosis of DOX-induced cardiotoxicity is essential, as once patients develop advanced HF, their prognosis is poor.^3^

Mechanistically, reactive oxygen species (ROS) and oxidative stress have been linked to DOX-induced cardiotoxicity^4, 5^ and could explain the relative tissue specificity of the adverse effects of DOX as the heart is rich in mitochondria, metabolically active and has relatively low antioxidant-producing enzymes.^6^ Others have highlighted the cardioprotective effects of cardiomyocyte-specific Top2*β* deletion, which suggest this enzyme could mediate the pathological side effects of DOX.^7^

Alterations in myocardial responses to catecholamines are well described in HF. Physiologically, *β*-adrenoceptor (*β*AR) activation by catecholamines results in protein G (G_s_)-dependent activation of adenylyl cyclase (AC), enhancing cyclic adenosine monophosphate (cAMP) production from ATP. cAMP activates protein kinase A (PKA), which is responsible for phosphorylating several proteins involved in Ca^2+^ metabolism and contraction. Although *β*_1_AR is only able to interact with G_s_, *β*_2_AR can bind both to G_s_ and to the inhibitory G_i_ protein; *β*_2_AR-G_i_ activation reduces contraction via both cAMP-dependent and -independent mechanisms. Chronic sympathetic activation is detrimental for the heart and sustained overexpression of *β*_1_AR triggers myocyte apoptosis and hypertrophy leading to HF.^8^ Sustained *β*-adrenergic stimulation leads to reduced expression and/or compensatory desensitization of *β*ARs to catecholamines, both features of the functionally impaired heart.^9^ Increased expression of G_i_—particularly of the *α*-2 subunit (G_iα-2_)—is also observed in HF, where enhanced *β*_2_AR-G_i_ coupling triggers diminished basal contractility following receptor activation. However, the mechanisms underpinning Gi upregulation in chronic HF have remained unknown.^10, 11^ *β*-Adrenergic antagonists (*β*-blockers) constitute a critical clinical advance in the treatment of HF, being able to improve cardiac output.^12, 13^

Contributing to the developing molecular picture of multiple diseases, microRNAs (miRNAs) are a class of short (~22 nucleotides, nt) non-coding RNAs that play influential roles in the post-transcriptional ‘fine-tuning' of gene expression.^14^ miRNAs are often coded in clusters and transcribed as single transcriptional units, each forming a primary miRNA, which is then processed and gives rise to multiple mature miRNAs.^15, 16^ The miRNA classification into families groups together those miRNAs that share the same ‘seed region', which is the sequence necessary for target messenger RNA (mRNA) recognition and is usually located between nucleotides 2 and 8.^17^ miRNAs are implicated in heart development and function^18, 19, 20^ and specific miRNA signatures have been associated with certain cardiac pathologies.^21, 22, 23, 24^ Nonetheless, few studies have validated their functional impact. Although recent evidence describes the dysregulation of miRNAs across the heart upon DOX treatment,^25, 26^ a cardiomyocyte-specific miRNA signature of DOX-triggered alterations, or their consequences, is currently lacking.

We identified the DOX-induced cardiomyocyte miRNA alterations in various models of cardiac toxicity. We then proceeded to uncover the regulation of pathways implicated in heart disease by the miR-30 family (miR-30), which we found to be downregulated by DOX. This miRNA family is formed by four clusters, located in different genomic regions and organized in the following way in both rat and human: miR-30a; miR-30c-2; miR-30b and miR-30d; miR-30e and miR-30c-1 (http://www.mirbase.org). We report that miR-30 fine-tunes the *β*-adrenergic pathway by targeting *β*ARs and G_iα-2_, while also inhibiting the expression of the pro-apoptotic gene BNIP3L/NIX. Overall, gene expression regulation by miR-30 seems to be protective against the toxic effects of DOX in cardiac cells.

## Results

### DOX triggers miR-30 family downregulation in cardiomyocytes

**Figure 1:**
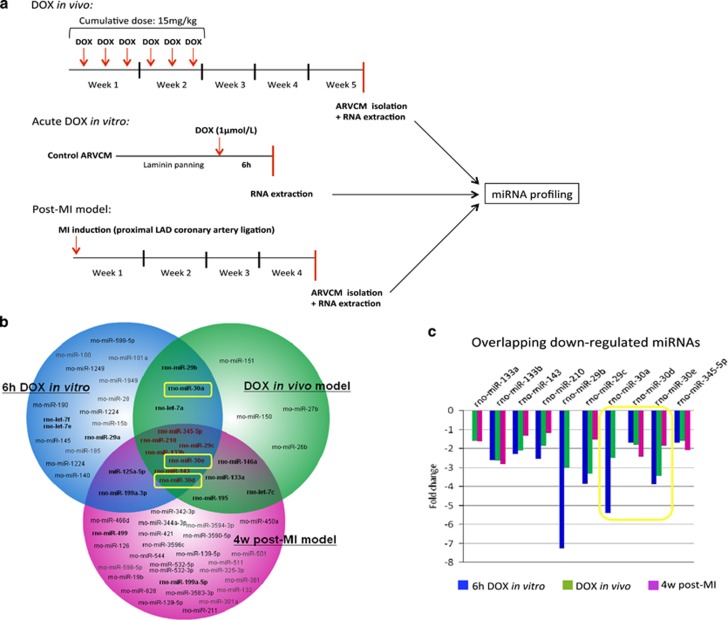
miRNA expression dysregulation occurs upon acute and sustained DOX treatment. (**a**) Experimental design of the models of study included in the miRNA profiling. DOX-induced HF *in vivo* model was generated by six i.p. DOX injections (2.5 mg/kg) spread over a fortnight. MI was induced by proximal LAD coronary artery ligation. For acute DOX treatment, cultured viable ARVCM were treated with 1 *μ*mol/l for 6 h. For all three models, RNA was isolated specifically from viable ARVCM. (**b**) Venn diagram showing significantly altered miRNA expression (>1.5-folds, *P*<0.05) detected for the profiled models of cardiac injury (*n*=3 per group). The three downregulated members of the miR-30 family are highlighted in yellow. Several other miRNAs have previously been linked to heart disease when dysregulated in the same direction as we observed and appear in bold writing. (**c**) miRNAs that presented reduced expression levels in at least two experimental models. rno, rattus norvegicus.

To obtain the miRNA signature of cardiomyocytes affected by DOX treatment, we established both *in vitro* and *in vivo* models. These included two different DOX treatments: an acute *in vitro* administration to primary isolated adult rat ventricular cardiomyocytes (ARVCMs) and an *in vivo* model of DOX-induced HF (Supplementary Figures 2 and 3), created by serial injections of the drug as previously described.^27^ An *in vivo* model of myocardial infarction (MI) was generated by proximal left anterior descendent (LAD) coronary artery ligation leading to pathological hypertrophy at 4 weeks and HF by 16–20 weeks and was included as reference of severe cardiomyopathy.^28^ A total of 26 miRNAs had significantly altered expression by ≥1.5-folds in DOX-treated cultured ARVCMs, 18 miRNAs in ARVCMs obtained from DOX-treated hearts and 40 miRNAs were dysregulated in ARVCM from infarcted hearts 4 weeks post MI. Seven miRNAs (miR-133b, miR-143, miR-210, miR-29c, miR-30d, miR-30e and miR-345-5p) were found to be downregulated in all three models (Figure 1). A subset of three overlapping hits (miR-30e, miR-210 and miR-29c) was chosen for validation by reverse transcription–quantitative PCR (RT-qPCR; Supplementary Figure 4). Moreover, sustained miRNA alteration during HF progression was confirmed by RT-qPCR at a later time of 16–20 weeks post MI stage (Supplementary Figure 5). We could also confirm aberrant expression of several miRNAs that had previously linked to heart disease in our models (Figure 1b, bold writing).^21, 22, 25, 29, 30, 31, 32, 33, 34, 35^ Strikingly, three members of the miR-30 family (miR-30a, miR-30d and miR-30e) were downregulated in at least two models, which indicated potential biological relevance. miR-30e showed the greatest dysregulation across all three models of study (Figure 1c).

### DOX induces *β*_1_AR, *β*_2_AR, G_iα-2_ and BNIP3L/NIX expression through miR-30 downregulation

**Figure 2:**
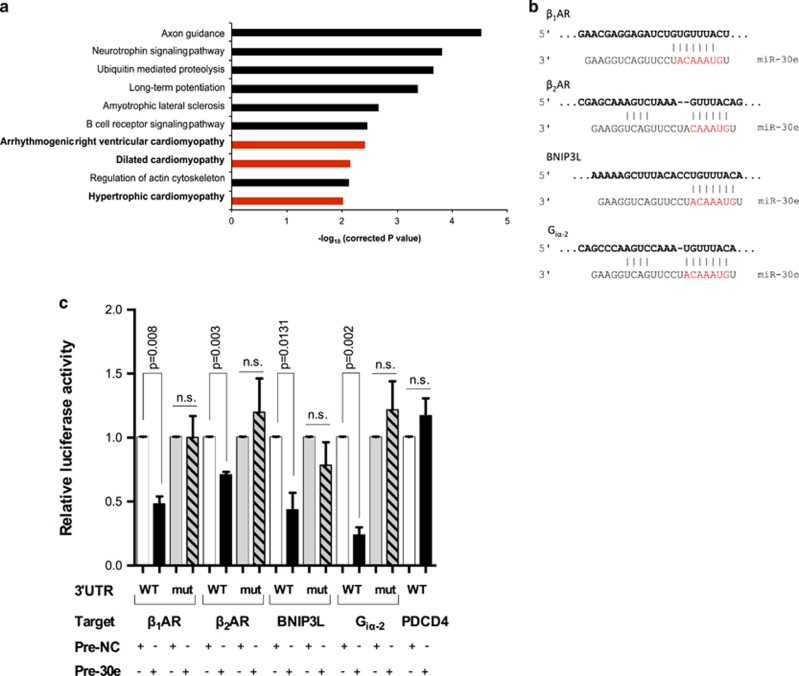
miR-30 target prediction and validation of direct 3′UTR regulation by 3'UTR luciferase assay. (**a**) Pathway enrichment analysis of the predicted miR-30 targets by TargetScan; performed using Database for annotation, visualization and integrated discovery. Three types of cardiomyopathy are part of the top 10 most significantly enriched pathways. (**b**) Representation of miR30e-mRNA annealing for four of the target genes predicted by TargetScan (*β*_1_AR, *β*_2_AR, BNIP3L and G_iα-2_). miRNA seed sequence is highlighted in red. (**c**) Relative luciferase activity measured on H9c2 cultures, after 24 h of co-transfection with either pre-30e or pre-NC (100 nmol/l) and the correspondent (wild type (WT) or mutant (mut)) 3′UTR luciferase reporter vector for each of the potential targets. The non-target programed cell death protein 4 (PDCD4) was included as negative control. Averaged (±S.E.M.) values are the result of at least three independent transfections (ns, not significant; *t*-test)

To further investigate the implication of miR-30 in DOX-induced myocardial damage, we used TargetScan software and retrieved a list of highly conserved predicted miR-30 target genes, based on seed pairing. Given that all family members share the same seed sequence, they are predicted to target the same genes.^36^ Pathway enrichment analysis of putative miR-30 targets using Database for annotation, visualization and integrated discovery (http://david.abcc.ncifcrf.gov/) revealed an implication of this target list in cardiovascular disease, being 3 out of the 10 most significantly enriched pathways related to cardiomyopathy (Figure 2a). Among the predicted miR-30 targets, we focused on four genes, which were identified as part of an interacting network by Cytoscape (Supplementary Figure 7) and thus considered high-confidence predictions. Three of these genes are involved in the *β*-adrenergic pathway: *β*_1_AR, *β*_2_AR and G_iα-2_. The fourth target selected was the BCL2/adenovirus E1B-interacting protein 3-like (BNIP3L or NIX), an important apoptotic effector capable of mediating mitochondrial death and triggering myocardial disease^37, 38, 39, 40^ (Figure 2b).

All four targets were first validated by luciferase assays using vectors containing the respective target 3'UTR downstream of the luciferase reporter gene (see Methods). Mutated versions of the 3'UTR sequences with disrupted miR-30-binding sites were also assayed to confirm specific miRNA–3'UTR interaction and the non-target programed cell death protein 4 (PDCD4) was included as additional negative control (NC). As cellular platform, we used H9c2 cardiac muscle cell line, which retains numerous features of mature cardiomyocytes and have been previously used to investigate DOX cardiotoxicity^41, 42^ (Supplementary Figure 6). A significant reduction in luciferase activity was recorded for all four predicted targets when co-transfecting with the corresponding 3'UTR vector and the miR-30e mimic (pre-30e) as representative of the family, compared with pre-NC. Conversely, as expected, no changes were observed when co-transfected with the mutated 3'UTR versions and for PDCD4 (Figure 2c). Overall, we demonstrated direct regulation of these four genes by miR-30.

**Figure 3:**
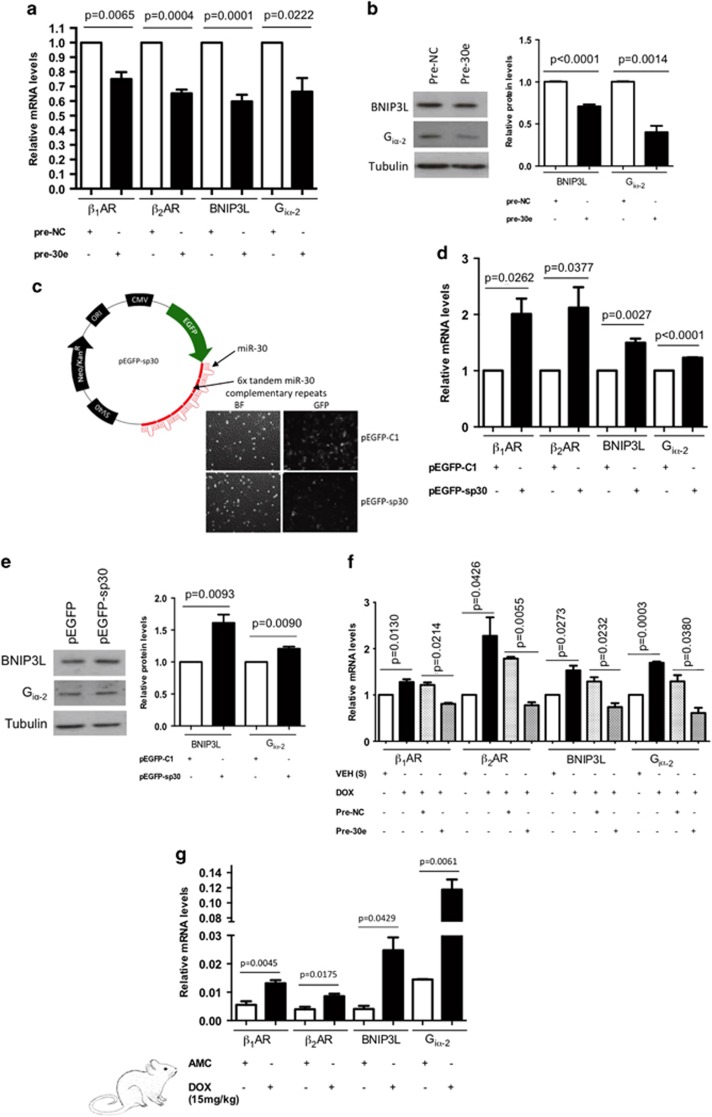
Experimental confirmation of *β*1AR, *β*2AR, BNIP3L and Gi*α*-2 as miR-30 targets. (**a**) RT-qPCR shows relative target expression levels when overexpressing miR-30. All values are normalized to U6 levels and presented as ratios to the pre-NC-transfected controls (20 nmol/l). Three biological replicates were performed on H9c2 cultures. (**b**) Relative protein levels of BNIP3L and G_i_*α*_-2_ were measured by western blot; band densitometry was quantified by ImageJ and normalized to tubulin. (**c**) Schematic view of the sponge vector designed for miR-30 family inhibition (pEGFP-sp30), using the pEGFP-C1 plasmid as backbone. EGFP expression in transfected cultures was detected by microscopy. (**d**) Relative target mRNA levels measured by RT-qPCR and protein levels (**e**) upon transfection with pEGFP-sp30 for miR-30 family inhibition, or the control vector pEGFP-C1. Data are averages of three biological replicates performed on H9c2 cardiac cells. (**f**) Target expression levels measured by RT-qPCR following DOX treatment (1 *μ*mol/l, 18 h), alone or in combination with miR-30e overexpression (pre-30e transfection). Three independent replicates were performed on H9c2 cells, being presented as averaged ratio to control ±S.E.M. (**g**) RT-qPCR data showing expression levels of the predicted miR-30 targets *in vivo* in DOX-treated hearts (15 mg/kg cumulative dose, *n*=5 rats per group). Student's t-test performed for statistical analysis. Error bars represent±S.E.M.

**Figure 4:**
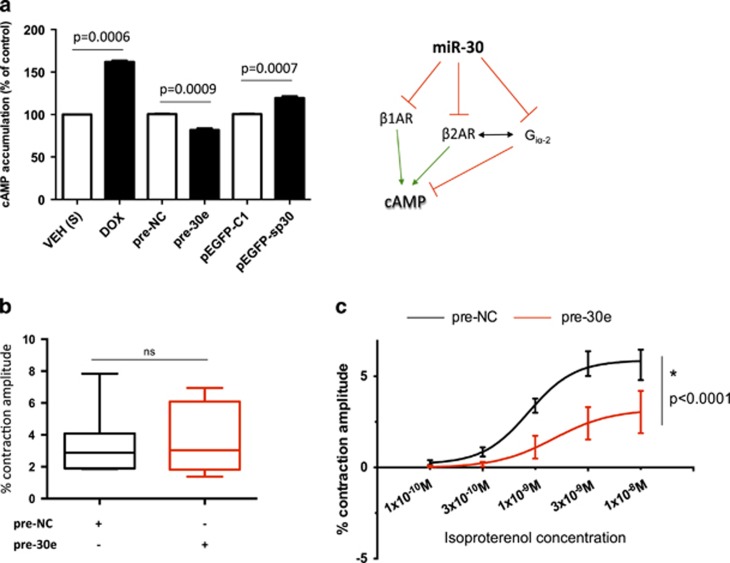
miR-30 effects on cAMP accumulation and contractility. (**a**) cAMP accumulation data expressed as % of the respective control samples. Values are the mean±S.E.M. of three independent experiments. The diagram on the right shows the contribution to cAMP production of the three miR-30 targets involved in the *β*-adrenergic pathway. (**b**) Baseline % of contraction amplitude of paced ARVCM transfected with either pre-30e or pre-NC for 48 h (100 nmol/l), measured using IonOptix (*n*=8–10 cells from 6 preparations; t-test; ns, not significant). (**c**) Contractile responses of isoproterenol-stimulated transfected ARVCM, recorded using IonOptix (*n*=6 cells from 6 preparations, F-test)

We next overexpressed miR-30e in H9c2 cardiac cultures and observed a decrease in target mRNA levels of the selected targets (Figure 3a). We quantified protein dysregulation of BNIP3L and G_iα-2_ by western blotting (Figure 3b) but this was not possible for *β*_1_AR and *β*_2_AR as there were no specific antibodies commercially available.^43, 44^ To partly overcome this issue, accumulation of cAMP was used as an indicator of *β*AR signaling, which validated miR-30 targeting (Figure 4a). Additional confirmation of miR-30-dependent regulation of target genes was achieved by miR-30 inhibition using an in-house sponge vector designed inhibit the entire miR-30 family^45^ (Figure 3c). In contrast to the results obtained upon miR-30e overexpression, increased target mRNA and protein levels were recorded (Figures 3d and e). These data were replicated independently using a commercial locked nucleic acid (LNA) construct for whole miR-30 family inhibition (Supplementary Figures 8 and 9). Remarkably, DOX treatment of H9c2 cardiac cells triggered an increase in miR-30 target mRNA levels, which could be fully reversed by simultaneous overexpression of miR-30 (Figure 3f). This indicates that mechanistically DOX is able to induce miR-30 target expression through miR-30 downregulation. Interestingly, miR-30 target upregulation also occurred *in vivo* upon DOX treatment, which is consistent with the demonstrated miR-30 downregulation in this disease model (Figure 3g). Together, these data suggest that *β*_1_AR, *β*_2_AR, G_iα-2_ and BNIP3L/NIX are novel miR-30 targets and that the downregulation of miR-30 by DOX is responsible for the increase in target gene expression.

### miR-30 expression attenuates the contractile response of cardiomyocytes to *β*AR stimulation

miRNAs can modulate the expression of hundreds of mRNAs and is the global regulatory effect on targets with opposing functions that will ultimately influence cellular phenotypes. After validating three members of the *β*-adrenergic pathway (*β*_1_AR, *β*_2_AR and G_iα-2_) as miR-30 targets, we evaluated the net effects of miR-30 dysregulation on this pathway. As mentioned, cAMP is a direct indicator of *β*AR activation while inversely correlates with G_i_ activity. We observed significantly increased cAMP generation upon acute DOX treatment, as well as following miR-30 inhibition with our pEGFP-sp30 vector. On the other hand, less cAMP accumulation was detected when overexpressing miR-30e in cardiac cultures (Figure 4a). These findings are consistent with the regulation of *β*ARs by miR-30 and suggest that miR-30 exerts a greater inhibitory effect on *β*ARs rather than G_iα-2_.

Having assessed the alterations that miR-30 expression modulation has on cellular cAMP, we wished to determine the global effects of miR-30 target regulation on cardiomyocyte contractility. We transfected primary isolated ARVCM with miR-30e mimics to achieve overexpression (Supplementary Figure 10) and recorded single-cell contractility using IonOptix. No significant changes were observed in the baseline percentage of contraction amplitude (Figure 4b). We then stimulated transfected ARVCM with increasing concentrations of isoproterenol, a *β*AR agonist, and analyzed paced contractile responses. Cells overexpressing miR-30e showed decreased contractile response to isoproterenol compared with control cells (Figure 4c), a finding supported by the cAMP accumulation data.

### miR-30 expression is protective against DOX toxicity

**Figure 5:**
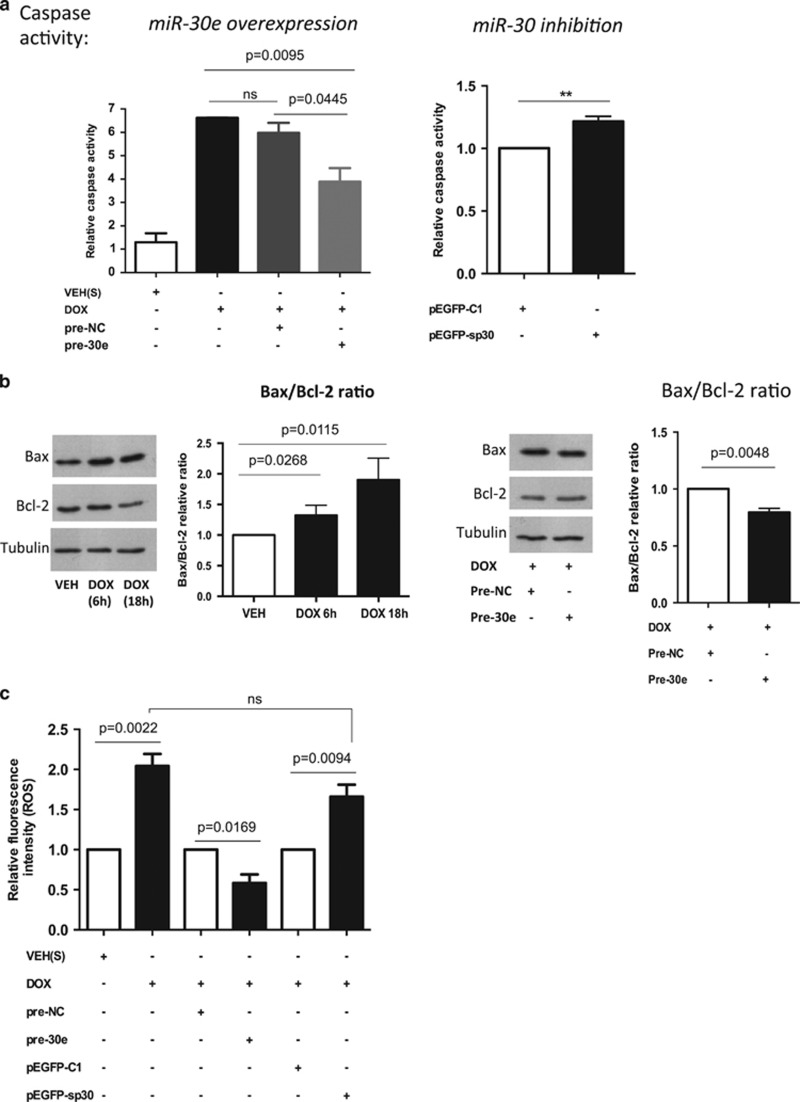
High miR-30 levels are protective against DOX insult and reduce ROS generation. (**a**) Left graph: caspase activity in cardiac cultures treated with DOX alone (1 *μ*mol/l, 18 h) and in combination with miR-30e overexpression through pre-30e mimic transfection (20 nmol/l). Right graph: relative caspase activity upon miR-30 family inhibition using the pEGFP-sp30 sponge vector. (**b**) Bax and Bcl-2 protein levels measured by western blot analysis. Cells were treated with DOX (1 *μ*mol/l, 6 h and 18 h) and also transfected with pre-30e mimics (20 nmol/l) in combination with DOX (1 μmol/l, 18 h). Band densitometry was measured using Image J. All values are normalized to Tubulin and expressed as Bax/Bcl-2 ratio. (**c**) ROS generation of transfected cultures for miR-30 overexpression or inhibition, measured by DCFDA fluorescence intensity. All assays (**a, b, c**) were performed on H9c2 cells and results are average of independent triplicates ±S.E.M. (*t*-test; ns, not significant)

It is known that DOX induces cellular toxicity mediated by caspase-dependent apoptosis.^46^ We investigated whether miR-30 expression modulation is sufficient to affect DOX-induced toxicity in cultured cardiac cells. We measured the activity of caspases 3 and 7 as apoptotic indicator. Ectopically increased miR-30e expression resulted in reduced apoptosis, bringing caspase activity closer to the levels observed upon vehicle treatment (Figure 5a). On the contrary, cultures presented higher caspase activity when inhibiting miR-30 with our pEGFP-sp30 sponge vector (Figure 5a).

DOX has also been described to increase the expression of Bax (pro-apoptotic gene) and to decrease Bcl-2 (anti-apoptotic gene) levels. Indeed, elevated Bax/Bcl-2 ratio increases susceptibility to death following apoptotic stimuli^47^ and restored Bcl-2 expression has been shown to protect cardiomyocytes against DOX toxicity.^48^ To further assess the protective effects of miR-30 against DOX insult, we measured the Bax/Bcl-2 expression ratio in treated cardiac cultures. As expected, DOX treatment progressively increased the Bax/Bcl-2 ratio. In contrast, miR-30e overexpression reduced the Bax/Bcl-2 ratio upon DOX treatment (Figure 5b). An additional characteristic of DOX-associated cardiomyocyte damage is high ROS levels.^49^ We therefore assessed the impact of miR-30 on ROS. Notably, cells with high miR-30e expression showed reduced ROS, whereas miR-30 inhibition resulted in higher ROS levels. No further increase in ROS production was observed when combining DOX treatment and miR-30 inhibition (Figure 5c). This finding further confirmed the benefit of high miR-30 expression in cardiac cells.

### GATA-6 mediates DOX-induced miR-30 downregulation

**Figure 6:**
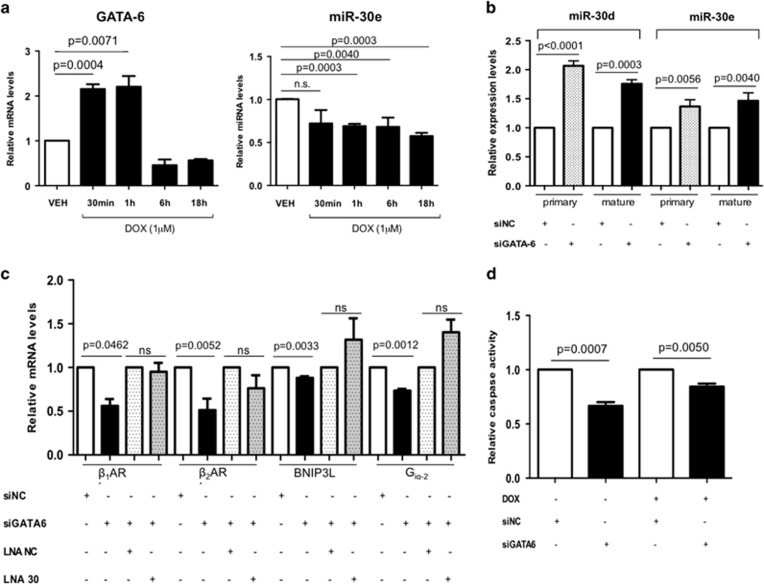
GATA-6 mediates miR-30 downregulation triggered by DOX. (**a**) RT-qPCR data showing GATA-6 and miR-30e expression levels in response to DOX time-course treatment (1 *μ*mol/l). (**b**) Relative levels of primary and mature miR-30d and miR-30e, upon GATA-6 silencing detected by RT-qPCR. (**c**) RT-qPCR data for miR-30 target mRNA levels in response to GATA-6 silencing (25 nmol/l, 72 h) and after co-transfection with compensatory LNA miR-30 family inhibitors (100 nmol/l, 72 h). (**d**) Relative caspase activity in response to GATA-6 silencing, ±DOX treatment (1 *μ*mol/l, 18 h). All values (**a, b, c, d**) were obtained performing biological triplicates on H9c2 cells and are presented as averaged ratio to the respective control±S.E.M. All RT-qPCR results were normalized to the correspondent U6 Ct value (ns, not significant; *t*-test)

We next wished to establish the mechanism via which DOX downregulates miR-30 expression. GATA-6 is a transcription factor highly expressed in the heart and known to play a key role in cardiac development.^50^ Interestingly, we discovered evidence of GATA-6 binding to miR-30 cluster promoters by data mining of chromatin immunoprecipitation sequencing experiments, using deepBase (http://deepbase.sysu.edu.cn/). Strikingly, we identified increased GATA-6 levels shortly (30 min–1 h) in primary isolated ARVCM after DOX treatment, accompanied by a sustained reduction in mature miR-30e expression from the 1 h time point (Figure 6a).

We silenced GATA-6 using RNA interference (Supplementary Figure 12) and, in order to unveil the nature of its regulatory effect, we quantified primary and mature miR-30 levels of two family members coded in different clusters (miR-30d, miR-30e) by RT-qPCR. GATA-6 inhibition resulted in increased levels of miR-30d and miR-30e, at both the primary and mature stages of their bioprocessing (Figure 6b), suggesting an inhibitory role for GATA-6 on the miR-30 promoters. Subsequently, we measured expression levels of the validated miR-30 targets (*β*_1_AR, *β*_2_AR, G_iα-2_ and BNIP3L) upon GATA-6 silencing, which were accordingly reduced (Figure 6c). Decreased target expression levels could be compensated by simultaneous miR-30 family inhibition using LNA oligonucleotides (Figure 6c), suggesting a direct mediation of the GATA-6 effects on target gene expression by miR-30. Finally, in order to determine the impact of GATA-6 on cell toxicity, we measured caspase activity in treated H9c2 cardiac cultures. GATA-6 inhibition was reduced activation of apoptotic pathways even in the presence of DOX administration (Figure 6d).

## Discussion

We describe here cardiomyocyte-specific alterations in miRNA expression following acute and chronic DOX injury, and post-MI induction. A shared miRNA subset was detected across the assayed models of cardiac toxicity, reflective of consistencies in the cardiomyocyte response to injury. However, those miRNAs only differentially expressed in response to acute and/or sustained DOX treatments also suggest the existence of unique molecular signatures in anthracycline-related myocardial damage (Figure 1b). We believe that the rapid alteration of these miRNAs within hours of exposure to DOX and maintained over time could be of translational relevance, both as diagnostic biomarker and as therapeutic strategy.

In particular, we describe here the myocardial downregulation of several miR-30 family members by acute and sustained DOX treatment and validate four essential genes for cardiomyocyte biology as novel miR-30 targets (*β*_1_AR, *β*_2_AR, G_iα-2_ and BNIP3L/NIX; Figures 2c and 3). These data are consistent with our previous findings in which miRNAs often target multiple members of a shared signaling pathway, as individual target inhibition *in vivo* is unlikely to be complete.^51^ Alterations in several of these genes have previously been linked to HF (i.e., desensitized *β*ARs, chronic *β*_1_AR activation and increased G_iα-2_ expression).^9, 10^ Moreover, the pro-apoptotic target gene BNIP3L/NIX has been linked to cardiomyopathy through mitochondrial cell death.^39, 52^ In concordance with the present data (Figure 3g), enhanced *β*_2_AR cardiac expression upon DOX treatment *in vivo* has recently been reported, although the mechanisms involved were not investigated.^53^ Moreover, *β*_2_AR deletion has been shown to prevent cardiomyopathy.^54^

Reduced miR-30 levels have been associated with myocardial matrix remodeling and with angiotensin II-induced hypertrophy.^31, 55^ Low miR-30e expression was also detected in myocardial tissue from patients with dilated cardiomyopathy and aortic stenosis.^56^ However, the specific myocardial expression of miR-30 in the context of exposure to DOX and its downstream mechanisms had not been previously demonstrated. We show that high miR-30 levels are connected to a global decrease in cAMP (Figure 4a), likely to be advantageous given the described link between high cAMP/PKA and cardiomyocyte death.^57^ The greater accumulation of cAMP triggered by acute DOX treatment than by specific miR-30 inhibition alone raises the hypothesis that DOX may be acting through alternate pathways toward the same detrimental effect. Upon miR-30e overexpression, ARVCM presented decreased contractile responses to isoproterenol stimulation. As *β*AR and G_iα-2_ have opposing effects on the same pathway, such attenuated contractile response reveals a phenotype suggesting preferential *β*AR repression alongside a compensatory inhibition of G_iα-2_ (Figures 4a and c, Supplementary Figure 11). This negative feedback loop is likely to contribute to the fine-tuning of the system, which is characteristic of miRNAs and allows intensive regulation of complex biological processes.^58^ The preferential *β*AR inhibition observed results in an overall effect simulating *β*-blocker agents, which are commonly used to treat HF. Furthermore, milder G_iα-2_ targeting by miR-30 could also tentatively be beneficial, as retention of G_i_ activity serves to reduce myocyte death.^59^

For the first time, we show that DOX treatment triggers an early induction of GATA-6 expression. In addition, our data suggest that DOX-induced GATA-6 upregulation represses miR-30 transcription, a model supported by (primary and mature) miR-30 upregulation in response to GATA-6 silencing. In keeping with this, miR-30-dependent target downregulation was observed upon GATA-6 inhibition (Figure 6).

Cardiomyocyte apoptosis, involving cytochrome c and caspase activation, is a crucial event in HF development in general and after DOX injury.^46, 60, 61^ Importantly, high miR-30 levels are protective against DOX insult while inhibition of miR-30 is sufficient to increase caspase-mediated apoptosis (Figure 5a). Consistent with this, the Bax/Bcl-2 ratio was lower in cardiac cultures overexpressing miR-30e upon DOX administration (Figure 5b) and ROS generation inversely correlated with miR-30 levels, further indicating the beneficial effects of miR-30 expression for cardiac cell viability (Figure 5c).

Interestingly, *β*-blockers have been shown to prevent cancer progression both in patient cohorts^62, 63^ and animal models.^64, 65, 66^ Moreover, increasing evidence indicates a tumor-suppressor role for miR-30, having been shown to minimize resistance to chemotherapy and to correlate with less invasive tumors.^67, 68, 69, 70^ We confirmed this finding in our system by Transwell migration assays performed on the highly metastatic breast cancer cell line MDA-MB-231, which had impaired migration upon miR-30 overexpression (Supplementary Figure 13). In addition, analyses of breast cancer patients' data freely available at the Gene Expression Omnibus (http://www.ncbi.nlm.nih.gov/geo/) consistently showed a reduction in miR-30 levels compared with control individuals (Supplementary Figure 14). Similarly, both SurvMicro (http://bioinformatica.mty.itesm.mx/SurvMicro) and PROGmiR (http://www.compbio.iupui.edu/progmir) prognostic databases indicated high miR-30 expression to be a biomarker of good prognosis in breast cancer (Supplementary Figure 15). These data are encouraging and suggest that controlled administration of miR-30 mimics could deliver a pleiotropic therapeutic effect. Unfortunately, we could not carry out the required experiments to investigate this further due to the restrictions applied on animal experimentation by the UK Home Office.

**Figure 7:**
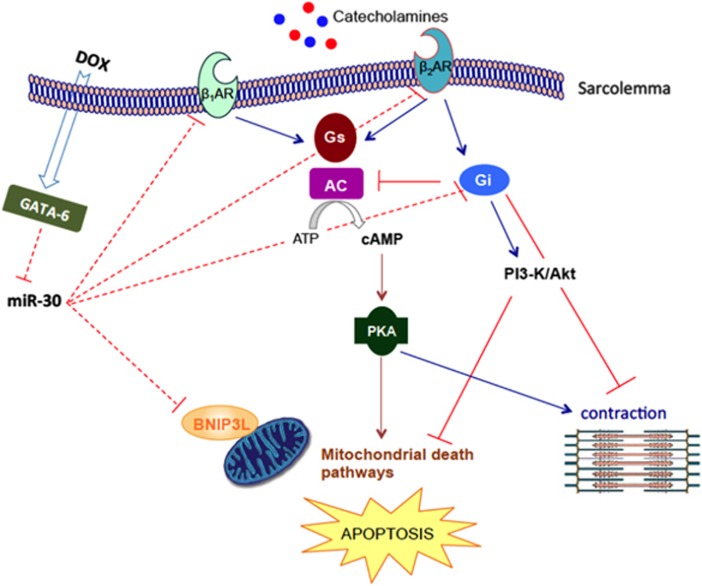
Proposed model: importance of the miR-30 regulatory effects in cardiomyocytes following DOX exposure. Upon DOX treatment, there seems to be an acute increase of GATA-6 expression in cardiomyocytes. GATA-6 is able to repress miR-30 family expression, resulting in DOX-induced miR-30 downregulation. In turn, the expression levels of miR-30 targets are upregulated. Three of the miR-30 target genes are involved in the complex catecholamine/adrenergic pathway. A correct equilibrium of members is crucial in this signaling cascade, as AC-cAMP-PKA activation promotes cardiomyocyte contraction, whereas Gi impairs contraction but protects against apoptosis via Phosphoinositide 3-kinase/Protein kinase B. In addition, miR-30 represses BNIP3L, which is involved in mitochondrial death. miR-30 expression seems to be essential in regulating mitochondrial apoptotic pathways and it is able to partially counteract DOX-induced toxicity. Solid lines: previously described mechanisms, dashed lines: novel mechanisms

In aggregate, our findings should contribute to unraveling the complex web of mechanisms via which DOX is able to cause cardiac toxicity and dysfunction, as we reveal the targeting of pathways involved in cardiomyocyte function and viability by a miRNA family that is altered by DOX (Figure 7).

## Materials and Methods

### Animal models

All animal surgical procedures and perioperative management were carried out in accordance with the United Kingdom Home Office Guide on the Operation of Animals (Scientific Procedures) Act 1986 by licensed personnel. See Supplementary Information for details of the experimental procedures.

#### MI induction

Adult male Sprague–Dawley rats (250–300 g) underwent proximal LAD coronary artery ligation to induce chronic MI as previously described by our group.^28^ Animals were anesthetized with 5% isofluorane in O_2_ and maintained on 1.5% isofluorane during surgery. 0.05% buprenorphine and 5 mg/kg enrofloxacil were administered intraperitoneally to prevent pain and infection. Two infarcted cohorts were generated. To model early post MI-hypertrophy, animals were killed 4 weeks after MI induction (*n*=5). Late-stage HF model hearts were explanted and prepared for cell isolation 16–20 weeks post MI surgery (*n*=5), when an advanced HF phenotype was established.^28^ Age-matched controls (AMCs, *n*=5 for each) were used in both cases to preserve animal welfare.

#### DOX-induced HF model

Adult male Sprague–Dawley rats (250–300 g) were administered a total cumulative dose of 15 mg/kg of DOX delivered via six intraperitoneal injections over 2 weeks (*n*=5). Each injection contained 2.5 mg/kg of DOX (Teva, Castleford, UK) diluted in saline up to a volume of 2 ml. AMC rats were used as controls (*n*=5). Animals were kept under constant monitoring for signs of HF and any other possible adverse effects. Three weeks after the last injection, animals were killed and hearts explanted, weighed and prepared for cell isolation.

### Ventricular cardiomyocyte isolation

Ventricular cardiomyocyte isolation from adult Sprague–Dawley rat hearts was carried out via serial enzymatic collagenase-hyaluronidase digestion as described previously^28^ (see Supplementary Information).

### Cell culture and treatments

Adult rat ventricular cardiomyocytes (ARVCM) were maintained in supplemented M199 (Invitrogen, Paisley, UK). ARVCM from AMC animals or DOX/4week post-MI models were plated in laminin (Invitrogen)-coated six-well plates and viable rod-shaped cells were allowed to attach for an hour. Apoptotic floating ARVCM were then discarded. For *in vitro* studies, medium was renewed and DOX (Teva) was then administered to cultured viable ARVCM. A clinically relevant concentration of 1 *μ*mol/l was used throughout.^6^ This dose was allowed to act in the cultures for 6 h for miRNA expression level check. For evaluation of gene expression changes (mRNA and protein), DOX treatments were conducted over 18 h. Control wells were treated with the same volume of vehicle (saline). H9c2 cardiac muscle cells were used as model to overcome the limitations of ARVCM,^41^ and were maintained in Dulbecco's modified Eagle's medium supplemented with 10% fetal calf serum, 1% penicillin/streptomycin and 2% L-glutamine.

### Transfections

H9c2 cells were plated at 40% confluence in six-well plates and transfected on the following day and for 72 h, with 20 nmol/l of precursor miRNAs for mimicry overexpression (pre-30e or pre-NC; Applied Biosystems, Paisley, UK) and 100 nmol/l of miRCURY LNA for family inhibition (Exiqon, Vedbaek, Denmark), using HiPerFect transfection reagent (Qiagen, West Sussex, UK). For sponge vector transfections (pEGFP-sp30 or pEGFP-C1 control), we used Lipofectamine 2000 (Life Technologies Ltd, Paisley, UK) on 90% confluent cultures and allowed them to get transfected for 48 h in antibiotic-free medium. For luciferase assays, H9c2 cells were plated at a density of 50 000 cells/well in 24-well plates and co-transfected the following day with 100 nmol/l of pre-30e/pre-NC and 100 ng of the respective pLightSwitch_3′UTR GoClone for each target 3'UTR, also using Lipofectamine 2000. In each independent replicate, three wells were transfected for each condition. For contractility studies, primary isolated ARVCM were transfected in six-well plates and kept in solution for 48 h. We used Lipofectamine 2000 (Life Technologies Ltd) and a molarity of 100 nmol/l of pre-30e or pre-NC.

### Sponge plasmid construction

The pEGFP-sp30 sponge vector was manufactured by annealing, purifying and cloning primers coding for six bulged tandem repeats of mismatched miR-30-binding sites. The insert was cloned into the pEGFP-C1 plasmid (Clontech, Mountain View, CA, USA) using the *Hin*dIII and *Bam*HI restriction sites, as 3'UTR of the EGFP-coding sequence. The generated sponge vector was then verified by sequencing. Primers used for sponge vector construction are described in Supplementary Table 1.

### miRNA profiling

miRNA profiling was performed using nCounter Rat miRNA Expression Assay (NanoString Technology, Seattle, WA, USA), using 150 ng of total RNA. Biological triplicates were assayed for all conditions. Background removal was performed following the producer's instructions and quantile normalization of raw data was carried out using Partek Genomic suite (St. Louis, MO, USA). A list of the 20 most significantly dysregulated hits for each model including *P* values and fold changes can be observed in Supplementary Table 3. The full list of assayed miRNA sequences and complete raw data has been deposited at Gene Expression Omnibus (GSE36239).

### 3′UTR Luciferase assays

After 24 h of co-transfection, cells were lysed using passive lysis buffer (Promega, Madison, WI, USA) and luciferase activity was detected using LightSwitch Assay System reagent (Switchgear Genomics, Menlo Park, CA, USA), in a GLOMAX 96 Microplate luminometer (Promega). The 3'UTR-Luc vectors used were *β*_1_AR (S812620), *β*_2_AR (S804637), BNIP3L (S812956) and G_iα-2_ (S807754), all from Switchgear Genomics. For 3'UTR site-directed mutagenesis of these vectors, we used the QuickChange II or II XL Site-Directed Mutagenesis Kit (Agilent Technology, Edinburgh, UK) according to the manufacturer's instructions. The sequences of the plasmid used for this protocol are reported in Supplementary Table 2. All resulting plasmids were verified by sequencing.

### cAMP accumulation

Treated H9c2 cells were lysed using 0.1 mol/l HCl. The lysates were then assayed using the 96-well strip cAMP EIA kit (Cayman Chemical, Ann Arbor, MI, USA) and analyzed according to the manufacturer's instructions. At least duplicate wells were included for each sample to account for technical variability.

### Caspase assays

Apoptosis in treated H9c2 cultures was measured by quantifying caspase 3 and caspase 7 activities, according to the manufacturers' instructions (Caspase-Glo 3/7 Assay, Promega). Briefly, both medium and cells were collected from each well and 50 *μ*l of cell suspension were transferred to a 96-well plate in duplicate. 100 *μ*l of Caspase-Glo reagent were then mixed into each well an incubated for 1 h at room temperature, before reading luminescence intensity using a PHERAstar^Plus^ machine (BMG Labtech, Aylesbury, UK). Luminescence value for caspase activity was normalized to protein content.

### ROS detection

Levels of ROS generation in the cultures were measured by using the MitoScience DCFDA Cellular ROS Detection Assay Kit (Abcam Plc., Cambridge, UK), according to the manufacturer's instructions. Fluorescence readings were performed in 96-well microplates, using a PHERAstar^Plus^ machine.

### Contractility of transfected ARVCM

Following 48 h of pre-miR-30e/NC transfection, ARVCM in suspension were transferred into a Perspex bath using a glass coverslip as base. Cells were superfused with KH solution (1 mmol/l Ca^2+^) at 37  °C, equilibrated with 95% O_2_/5% CO_2_. An inverted microscope was used for imaging and myocytes were electrically stimulated at 0.5 Hz, measuring cell shortening with the IonOptix system. Values were presented as % contraction amplitude. Cardiomyocytes from six different preparations were used for each treatment.

### Statistical analysis

Unless differently stated, data are presented as mean±S.E.M. calculated using Prism 5 (GraphPad Software Inc.) and Student's *t*-test was used for value comparison, being *P*<0.05 considered as significance threshold. To analyze the contractile responses of ARVCM to increasing concentrations of isoproterenol, F tests were performed.

## Abbreviations

*β*AR: *β*-adrenergic receptor
AC: Adenylyl cyclase
AMC: Age-matched control
AR: Adrenoceptor
ARVCM: Adult rat ventricular cardiomyocytes
Ca^2+^: Calcium
cAMP: 3',5'-Cyclic adenosine monophosphate
DOX: Doxorubicin
Gi: Guanine-coupled inhibitory protein
Gs: Guanine-coupled stimulatory protein
GTP: Guanine triphosphate
HF: Heart failure
LAD: Left anterior descendent
LNA: Locked nucleic acid
MI: Myocardial Infarction
miRNA: microRNA
mRNA: Messenger RNA
NC: Negative control
PI3-K: Phosphoinositide 3-kinase
PKA: Protein kinase A
ROS: Reactive oxygen species
RT-qPCR: Reverse transcription–quantitative PCR
