# Supplementary material for: Myocardial MiR-30 downregulation triggered by doxorubicin drives alterations in β-adrenergic signaling and enhances apoptosis
Source: Cell Death Dis. 2015 May 7;6(5):e1754–. doi: 10.1038/cddis.2015.89 (PMC4669718; doi:10.1038/cddis.2015.89)
Supplement: Supplementary Tables [file cddis201589x1.docx]

**SUPPLEMENTARY INFORMATION**

Supplementary experimental procedures

**RT-qPCR**

The reverse transcription (RT) of mature miRNAs was performed using 10ng of total RNA and the TaqMan MicroRNA Reverse Transcription Kit (Applied Biosystems). 1.5µL of the miRNA-specific TaqMan probe were combined with 6µL of RNA plus master mix. For gene expression quantification, the RT step was carried out using the Superscript III Reverse Transcriptase (Invitrogen) and the reverse primer of the couple designed to expand an exon-exon region. 0.5-1µg of total RNA was firstly mixed with 50nM of the reverse primer and incubated at 70°C to allow RNA denaturation and primer annealing. Master mix was then added to each tube, reaching a volume of 10µL. Reactions were performed by incubating samples in a 7900Ht Thermal Cycler (Applied Biosystems), following the protocol specified by the provider in each case. Once cDNA (complementary DNA) synthesis was completed, samples were placed on ice, resuspended in an appropriate volume of H2O and prepared for quantitative PCR.

For miRNA expression level determination, 1ng of the obtained cDNA was mixed with the appropriate volume of TaqMan Universal PCR Master Mix and 20X real-time probes (both Applied Biosystems) in a Fast Optical 96-well plate. Each sample was assayed in duplicate and the small nuclear (sn)RNA U6 control was included throughout. For gene expression (mRNA) levels, the transcripts of interest were amplified using SYBR Green (Applied Biosystems) and the correspondent forward/reverse primers (Sigma). Three wells of a Fast Optical 96-well plate were included per sample as technical replicates. All values were normalized to (sn)RNA U6. For both TaqMan and SYBR Green protocols, a final volume of 20µL/well was reached (5µL cDNA dilution plus 15µL of qPCR master mix). Plates were sealed with Optical Adhesive Covers (Applied Biosystems) and centrifuged briefly to ensure that the entire volume was at the bottom of the wells. qPCR was performed on an ABI Prism 7900HT sequence detection system (Applied Biosystems) and 40 amplification cycles were programed. Finally, data was analyzed using qBasePlus software (Biogazelle).

**Ventricular cardiomyocyte isolation**

In brief, rats were injected with 1000U/kg of heparin (Wockhardt, Wareham, UK) (i.p.) prior to killing, in order to prevent thrombosis. Hearts were dissected out rapidly and immersed in ice-cold Krebs solution, previously bubbled with 95% O_2_/5% CO_2_. The aorta was then cut below the bifurcation point and cannulated into a Langendorff perfusion apparatus, maintained at 37˚C. Hearts were first perfused with Krebs solution for 5 minutes. Low calcium medium gassed with 100% O_2_ was then perfused for further 5 minutes. Finally, the hearts were perfused with an enzyme solution, gassed with 100% O_2_, containing 1mg/mL of collagenase (Cooper Biomedical Inc., Malvern, USA) and 0.6mg/mL of hyaluronidase (Sigma Aldrich Ltd., Dorset, UK) [C&H solution], which was collected and re-circulated for 10 minutes. Hearts were then detached from the cannula and the atria were removed. Ventricles were cut into strips and transferred into a falcon tube with C&H solution. Tissue pieces were shaken and oxygenated for 5 minutes. After recovery of isolated cells, this step was repeated on the remaining tissue. Cell suspensions were centrifuged at low speed (40*G*) for 1 minute in order to pull down exclusively large cells (cardiomyocytes), confirmed by microscopic visualization. Cell pellets were then resuspended and isolated cardiomyocytes were finally spun and washed three times with DMEM before culturing in M199 medium (supplemented with: 1% P/S, 2g/L BSA, 0.0176g/L Ascorbic acid, 0.66g/L Creatine, 0.626g/L Taurine, 0.3224g/L Carnitine).

**Calcium transient measurements**

Freshly isolated cardiomyocytes were loaded with 10µmol/L of cetoxymethyl ester (AM) indo-1 Ca^2+^-sensitive dye (Invitrogen, Paisley, UK) at room temperature during 25 minutes. The dye was allowed to be de-esterified in the cells for 30 minutes. Cardiomyocytes were then transferred to a recording chamber superfused with Tyrode’s solution (in mmol/L: NaCl 140, CaCL2 2, KCL 6, MgCl 1, HEPES 10 and glucose 10) on plates coated with mouse laminin (Sigma Aldrich, UK). A stimulation field of 0.5Hz at a pulse length of 2ms was applied. Video cellular edge detection allowed contraction monitoring, and concomitant cytoplasmic calcium transients were recorded upon excitation of indo-1AM.

**Sarcoplasmic reticulum calcium release studies**

Ca^2+^ content in the SR was assessed by measuring Ca^2+^ transients produced by a rapid application of 10mmol/L of caffeine. Cardiomyocytes were subjected to normal stimuli applied at 0.5 Hz prior to the caffeine exposure.

**Sarcoplasmic reticulum calcium leak analysis**

Total SR leak was measured using the tetracaine-dependent SR leak. Cardiomyocytes were stimulated at 1Hz for at least three minutes in order to achieve a steady SR Ca^2+^ load. Stimulation was then stopped and the solution switched from NT to a 0Na^+^0Ca^2+^ solution (Li replacement and 1mmol/L EGTA) with or without tetracaine (1mmol/L, Sigma Aldrich, UK) for 30 seconds. 0Na^+^0Ca^2+^ was used to prevent movement of Ca^2+^ across the sarcolemma. Changes in cytosolic Ca2+ that occur in 0Na^+^0Ca^2+^ reflect movement of Ca^2+^ between the SR and the cytosol. SR load measured by applying 20mmol/L caffeine in 0Na^+^0Ca^2+^for approximately 3 seconds at the end of the tetracaine step.

***In vivo* pressure-volume analysis**

Pressure-volume (PV) analysis of left ventricular function was performed under general anaesthesia (1.5% isoflurane) using a 1.9F Scisense PV catheter attached to the ADVantage acquisition system (Scisense Inc., Ontario, Canada). Steady state and dynamic (IVC occlusion) studies were performed followed by rapid explantation of hearts for cardiomyocyte isolation. Data analysis was performed using PVAN 3.6 software (Millar Instruments, Texas, USA).

**Cardiomyocyte calcium studies**

Ventricular cardiomyocytes were loaded with the calcium indicator fluo4AM and field-stimulated calcium transients were recorded using Confocal Linescan microscopy as previously described ([1](#_ENREF_1)). Sarcoplasmic reticulum (SR) calcium stores and leak were measured using caffeine and tetracaine application respectively as previously described ([1](#_ENREF_1)).

**Transwell migration assays**

MDA-MB-231 cells were maintained in accordance to ATCC recommendations. Transfection of precursors (pre-30e and pre-NC, 20nM) and of sponge vector (pEGFP-sp30 or pEGFP-C1 control) was performed exactly as described for H9c2 cultures. Cells were trypsinized after transfection and 50,000 cells were plated on each membrane insert with 8mm pores (BD Biosciences), fitted into 24-well plates. Medium used for plating was serum-free, and cells were allowed to migrate towards complete growth medium through the porous membranes prior to staining and visualizing. Migrating cells per field were quantified using ImageJ software.

**Table S1. Primers used for RT-qPCR and sponge vector construction**

**Table S2. Primers used for site-directed mutagenesis of 3’UTR regions**

DOX-induced cardiomyopathy model

The phenotype of DOX-treated hearts was characterised. *In vivo* DOX administration induced a dilated cardiomyopathic phenotype with evidence of reduced systolic function. The cellular phenotype was consistent with significant heart failure, with prolonged calcium transient decay and increased calcium leak from the internal sarcoplasmic reticulum store (*Figures S2-S3*).

**
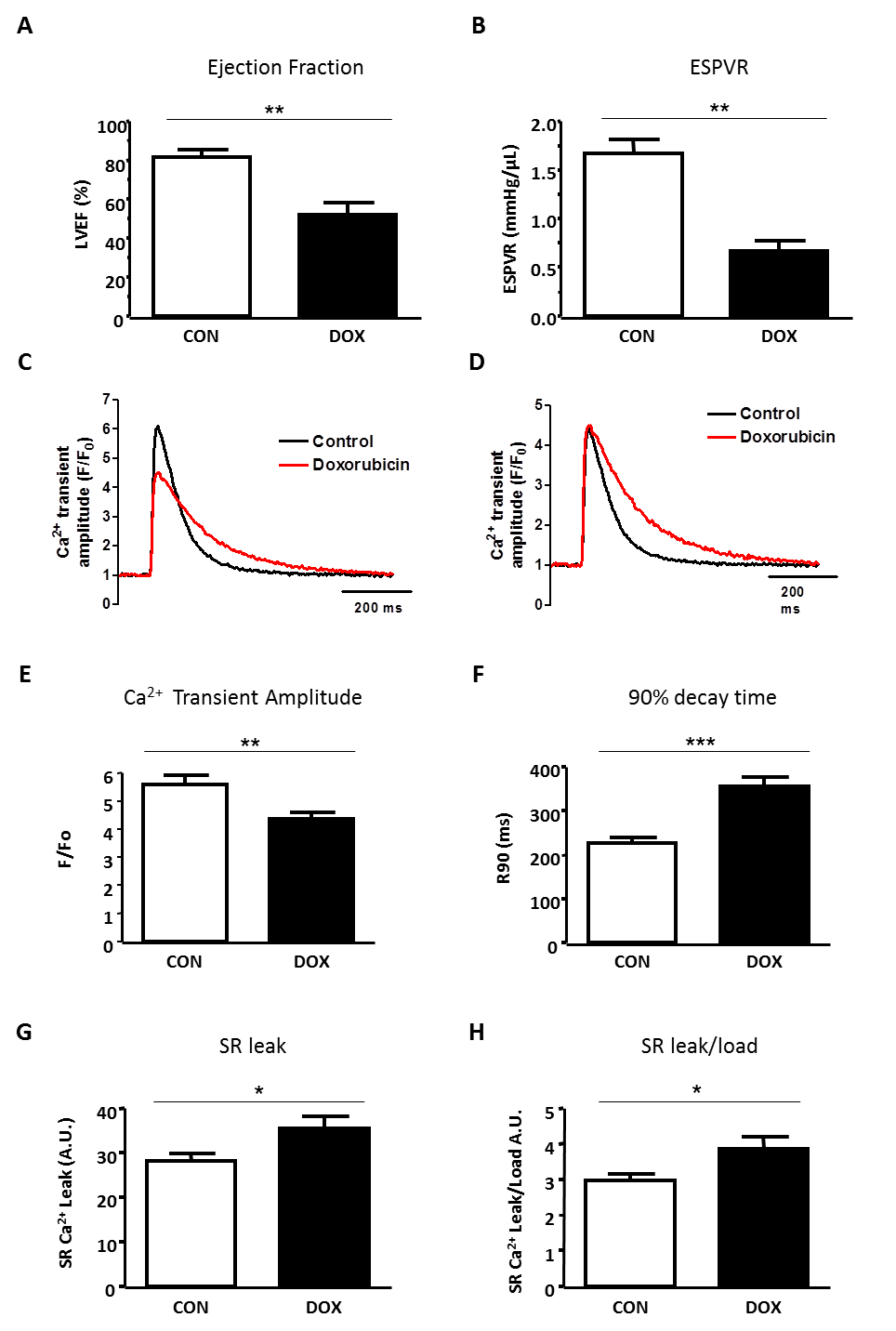
**

**Figure S2. Doxorubicin-induced cardiomyopathy.** DOX injection *in vivo* induced a dilated cardiomyopathic phenotype after 3 weeks, with reduced left ventricular systolic function measured using pressure-volume analysis. Left ventricular ejection fraction **(A)** and end systolic pressure-volume relationship **(B)** were significantly reduced (Control (CON) n=8, DOX n=3). **C.** Representative cytoplasmic calcium transient tracings from field stimulated (0.5Hz) isolated rat cardiomyocytes demonstrating reduced amplitude and prolonged relaxation in cardiomyocytes from DOX-treated hearts (red) compared to healthy controls (black). **D.** Overlain amplitude-matched transients demonstrate the prolonged decay in cardiomyocytes from DOX-treated hearts. Quantitative comparison of calcium transient amplitude **(E)** and clearance (time-to-90% decay (R90)) **(F)** in cardiomyocytes paced at 0.5Hz (CON n=23, DOX n=18). Tetracaine-sensitive SR calcium leak **(G)** and SR calcium leak corrected for SR calcium load **(H)** was increased in cardiomyocytes from the DOX-treated hearts, consistent with a failing phenotype (CON n=26, DOX n=18) (*p<0.05, **p<0.01, ***p<0.001).

**
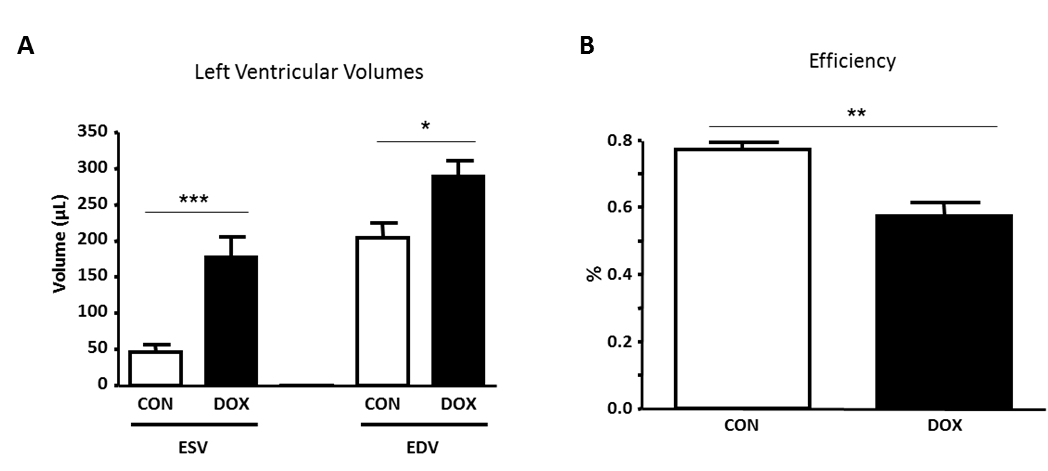
**


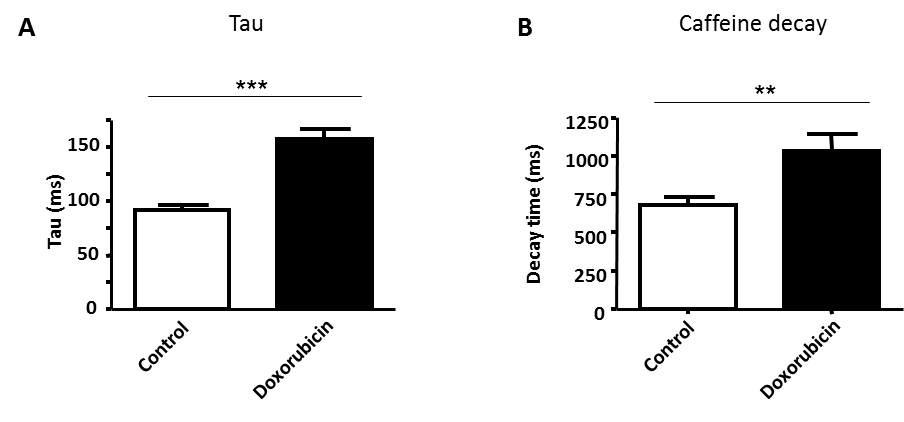


**C**

**D**

**Figure S3. Left ventricle volumes, efficiency of cardiac contraction and cytoplasmic calcium clearance. A.** DOX-treated hearts show increased end systolic volume (ESV) and end diastolic volume (EDV) consistent with a dilated cardiomyopathy phenotype. **B.** Contraction Efficiency (stroke work area/total pressure volume area) is reduced after DOX administration *in vivo*. **C.** Decay constant (Tau) of stimulated cytoplasmic calcium transient (0.5Hz). **D.** Decay time in the absence of Na^+^ and Ca^2+^ following total SR calcium release after caffeine application is prolonged in cardiomyocytes from DOX-treated hearts (*p <0.05, **p <0.01, ***p <0.001).

First 20 significantly dysregulated miRNAs for each of the profiled conditions

**Table S3. 20 most significantly dysregulated miRNAs as detected by profiling.** P values and fold changes detected for miR-30 in all three models highlighted in yellow. Primary isolated rat cardiomyocytes were treated with DOX *in vitro* for 6h at 1μM. For *in vitro* treatments, rats were injected with a cumulative dose of 15mg/kg and sacrificed 3w after the last injection. MI was induced by proximal coronary artery ligation; animals were sacrificed 4w post-surgery.

For complete record of Nanostring miRNA nCounter® raw and normalised data for this experiment see GEO website (accession number: GSE36239).

Experimental validation of Nanostring nCounter miRNA signature profiling

We chose three miRNAs detected to be altered across all models for RT-qPCR validation. The overlapping down-regulation of miR-29c, miR-210 and miR-30e observed in the profiling was reproducible by RT-qPCR (Figures S4 A-C). No alterations in miR-17-5p expression, which was unchanged across sample groups in the microarray, were detected in the RT-qPCR validation, thus acting as negative control (Figure S4 D). Hence, our RT-qPCR results correlate with those from the profiling (Figure 1), suggesting that the microarray experiment is valid and reliable. Subsequently we evaluated whether miRNA deregulation and its directionality was maintained longer after MI induction, in a model of late stage HF. Pure cardiomyocytes were obtained from rats 16-20 weeks after undergoing coronary artery ligation. Relative miRNA levels were measured by RT-qPCR in comparison to age-matched controls (AMC)**.** Importantly, these results show that the deregulation observed by the microarray at 4 weeks post-MI is conserved at a later stage (Figure S5).

**Figure S4. RT-qPCR validation of the microarray profiling results. A.** No significant expression changes were detected for miR-17-5p, which did not change across sample groups in the microarray profiling, by RT-qPCR **B.** miR-29c expression levels match those detected by microarray across all sample groups, showing down-regulation in all three models of cardiac toxicity (* p<0.05). **C.** miR-210 down-regulation also correlates with the profiling data (* p<0.05, ** p<0.001). **D.** miR-30e relative levels detected by RT-qPCR show significant down-regulation after DOX treatment *in vitro* (* p<0.05) and also in both *in vivo* models (** p<0.01) [S: saline, AMC: age-matched control].

**Figure S5. Changes in miRNA expression are maintained in a model of late stage HF.** RT-qPCR reveals reduced miR-29c, miR-210 and miR-30e expression levels in the 16w post-MI late stage HF model in relation to age-matched controls (AMC). All values normalized to U6 Ct and expressed as ratio to AMC expression (**p<0.005, ****p<0.0005).

miR-29c, miR-210 and miR-30e are down-regulated by DOX in H9c2 cells

**Figure S6. DOX down-regulates the expression of miR-29c, miR-210 and miR-30e in H9c2 cells. A.** RT-qPCR data showing miRNA expression (miR-29c, miR-210, miR-30e) relative to U6 levels for untreated primary isolated ARVCM and H9c2 cardiac cell line. All values expressed as ratios to U6 expression. **B.** Effects of DOX treatment (6h, 1μM) in the expression of the miRNA chosen for microarray validation (miR-29c, miR-210, miR-30e) on H9c2 cells as quantified by RT-qPCR. Values of independent triplicates are normalized to U6 and expressed as ratio to control [VEH(S): saline]. (*p<0.05, **p<0.005).

Cytoscape interactome analysis of the predicted miR-30 targets by TargetScan


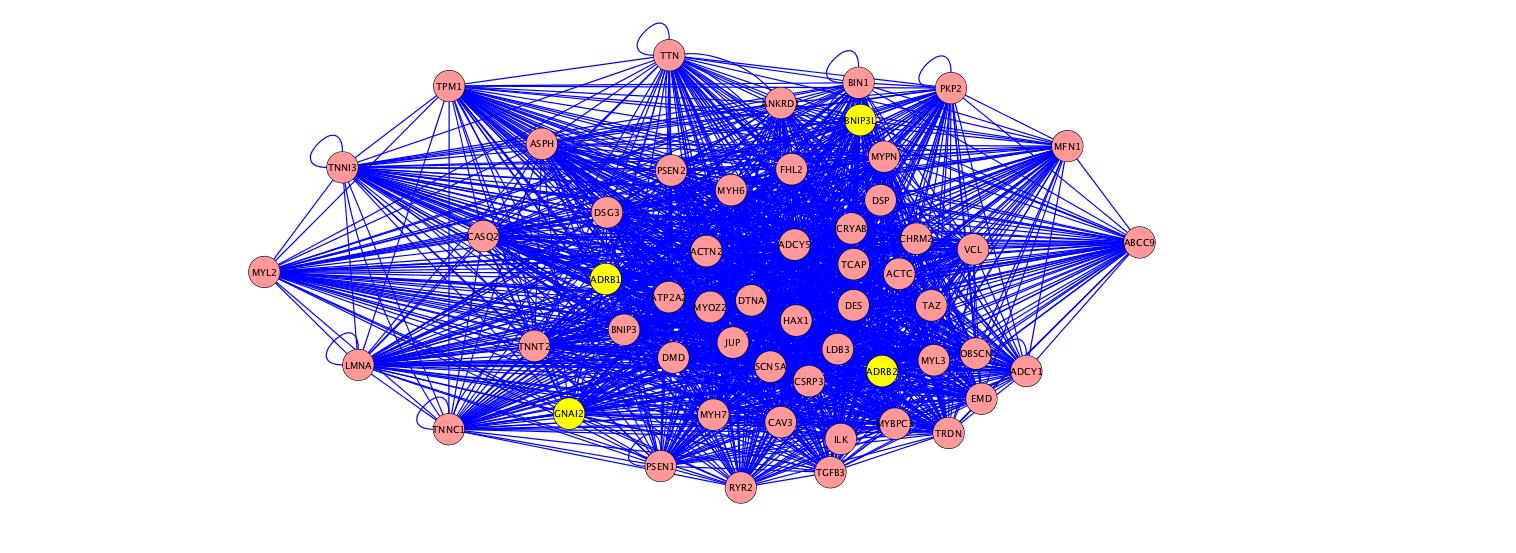


**Figure S7. Gene interaction network for the predicted miR-30 targets by TargetScan.** Graphic view of genetic interactions generated using Cytoscape. Nodes highlighted in yellow (ADRB1, ADRB2, BNIP3L and GNAI2) were selected for further investigation.

Evidence of inhibitory activity of the pEGFP-sp30 vector and commercial LNA30 oligonucleotides

**Figure S8. Mature miR-30e levels are reduced by both pEGFP-sp30 and LNA30. A.** RT-qPCR data showing mature miR-30e quantification as averaged ratio of three independent transfections. All Ct values were normalised to U6 expression (** p<0.005).

Target validation: miR-30 inhibition using commercial LNA oligonucleotides

Commercial LNA oligonucleotides directed against the whole miR-30 family were used to replicate and further validate the results obtained when using the homemade pEGFP-sp30 vector for miR-30 inhibition:

**Figure S9. miR-30 family inhibition by LNA results in comparable effects to pEGFP-sp30 treatment. A.** Target mRNA are increased in response to miR-30 inhibition by LNA oligonucleotide. **B.** Increased levels of cAMP accumulation correlate with βAR inhibition by miR-30. **C.** Protein level increase for BNIP3L and G_iα-2_. **D.** Increased caspase activity in the cultures where miR-30 is ablated by LNA. (*p<0.05; **p<0.01; *** p<0.001).

Confirmation of miR-30e overexpression in transfected ARVCM

Cardiomyocytes are a difficult cell type to transfect. In order to increase transfection rate, Lipofectamine 2000 was used instead of HiPerfect for miR-30e overexpression, and the concentration of mimics (pre-30e and pre-NC) was increased in comparison to transfections performed on H9c2 cultures. miR-30e overexpression was confirmed by RT-qPCR:

**Figure S10. miR-30e overexpression in transfected ARVCM.** RT-qPCR data showing miR-30e levels achieved in ARVCM after 48h of transfection with 100nM of pre-30e or pre-NC using lipofectamine 2000. Values were normalized to U6 expression and expressed as a ratio to pre-NC control samples (*** p<0.001).

Primary isolated ARVCM contractility in response to miR-30 overexpression and Pertussis toxin (PTX) treatment

PTX treatment was performed to block Gi activity in transfected ARVCM (either with pre-30e or pre-NC). PTX inhibits the interaction between Gi and G protein-coupled receptors (GPCR), impairing its intracellular cascade.

Cultured transfected ARVCM were exposed to PTX (Sigma) at a 1.5µL/mL concentration for 3h at 37°C, as previously optimised by our collaborators ([2](#_ENREF_2)). Following this treatment, contractility in response to catecholamine (isoproterenol) stimulation was measured using the IonOptix system:

**Figure S11. Contractile responses of miR-30e overexpressing ARVCM to isoprenaline stimulation combined with PTX treatment. A.** % contraction amplitude induced by increasing concentrations of ISO on pre-30e or pre-NC transfected ARVCM (100nM, Lipofectamine 2000, 48h), treated with PTX (1.5μg/mL) 3h before contractility measurement (n=6, F test). **B.** Transfected ARVCM (as in A.) comparing PTX treatment to no treatment. (n=6, F test, n.s. non significant).

Confirmation of GATA6 silencing

**Figure S12. GATA-6 silencing by siRNA transfection. A.** Reduction of GATA-6 mRNA levels by a specific siRNA. Expression checked by RT-qPCR and normalised to U6 levels. **B.** Reduction of GATA-6 protein levels, using actin expression as normaliser. All values represented as mean ±SEM of three biological replicates performed on H9c2 cardiac cultures (*** p<0.001).

miR-30 implications in breast cancer

Transwell assays were performed using MDA-MB-231 breast cancer cell line in order to evaluate the net effects of miR-30 modulation on the migratory phenotype, characteristic of aggressive cancers *(Figure S12)*. To further evaluate the relevance of miR-30 expression in association with breast cancer, we proceeded to bioinformatically analyse freely available patients data deposited in GEO (Gene Expression Omnibus) ([3](#_ENREF_3)). The miRNA profiling study selected – GSE26659- compared 77 breast carcinoma biopsies to 17 relapse-free mammoplasty controls. Analysis of differentially expressed miRNAs between these two groups revealed reduced miR-30 expression in the breast carcinoma group, being only miR-30e and miR-30a family members statistically significant.

**Figure S13. miR-30 expression correlates with reduced breast cancer cell migration. A.** Transwell migration assays were performed on MDA-MB-231 cells following 72h of precursor transfection (20nM, pre-30e/pre-NC) or 48h of pEGFP-30/pEGFP-C1 (1μg per well in 6-well plates). 50.000 cells were transferred in triplicate to 24-wells containing the membrane inserts and cells were allowed to migrate towards the chemo attractant medium (FCS containing) below the insert for 9h. Inserts were then stained with crystal violet and 5 fields of view were evaluated under the microscope. Quantification of migrating cells is expressed as the mean±SEM % of the respective control condition (bar graph). Three independent replicates were performed, and 3 membranes were quantified each time. (*p<0.05, *p<0.001). **B.** RT-qPCR data indicating miR-30e expression in different MDA-MB-231 cell lines derived from different tumour stages/locations [MDA-MB-231 (origin), T MDA-MB-231 (from primary tumour), L MDA-MB-231 (from lung metastasis)].

**Figure S14. Reduced miR-30 levels correlate with breast cancer. A.** GEO2R analysis of GEO dataset GSE26659 revealed significant miR-30e and miR-30a down-regulation in breast cancer patients compared to the control cohort. **B.** Normalised miRNA levels (miR-30e and miR-30a) recorded for all patients of the breast cancer (BC) and control groups. (**p<0.01, ***p<0.001).

**Figure S15. High miR-30a and miR-30e expression indicate good prognosis in breast cancer. A.** Box plots showing miR-30a and miR-30e expression levels in the low risk and high risk groups of a 528-sample breast carcinoma cohort, as retrieved by SurvMicro. **B.** PROGmiR Kaplan-Meier survival curve of breast cancer patients with high (red) and low (green) miR-30a expression. p values indicated in the graphs.

**Supplementary references**

1. Lyon AR, Bannister ML, Collins T, Pearce E, Sepehripour AH, Dubb SS, et al. SERCA2a Gene Transfer Decreases Sarcoplasmic Reticulum Calcium Leak and Reduces Ventricular Arrhythmias in a Model of Chronic Heart Failure / Clinical Perspective. Circulation: Arrhythmia and Electrophysiology. 2011 June 1, 2011;4(3):362-72.

2. Gong H, Adamson DL, Ranu HK, Koch WJ, Heubach JF, Ravens U, et al. The effect of Gi-protein inactivation on basal, and β1- and β2AR-stimulated contraction of myocytes from transgenic mice overexpressing the β2-adrenoceptor. British Journal of Pharmacology. 2000;131(3):594-600.

3. Gene Expression Omnibus <http://www.ncbi.nlm.nih.gov/geo/>. Available from: <http://www.ncbi.nlm.nih.gov/geo/>.
